# Case Report: Massive emphysematous pyelonephritis with short-term relapse

**DOI:** 10.3389/fmed.2025.1660972

**Published:** 2025-10-20

**Authors:** Tingfei Jiang, Letian Meng, Tong Chen, Jie Yang, Xiao Li, Liangyu Yao, Da Zhong, Ninghong Song

**Affiliations:** ^1^Department of Urology, The Affiliated Cancer Hospital of Nanjing Medical University and Jiangsu Cancer Hospital and Jiangsu Institute of Cancer Research, Nanjing, China; ^2^Department of Urology, The First Affiliated Hospital of Nanjing Medical University, Nanjing, China; ^3^The Second Affiliated Hospital of Wenzhou Medical University, Wenzhou, China

**Keywords:** emphysematous pyelonephritis, relapse, therapy, prognosis, infection

## Abstract

**Background:**

Emphysematous pyelonephritis (EPN) is a rare but aggressive infectious disease that lacks specificity in its early stages and tends to progress to life-threatening septic shock in a short period of time. Its diagnosis is based on imaging tests, and treatment requires a combination of staging and risk factors.

**Case presentation:**

We report a case of emphysematous pyelonephritis in a patient with massive emphysema of the left kidney (the longest diameter up to 51 mm) on CT. After multidisciplinary consultation, the patient underwent CT-guided percutaneous drainage combined with targeted antibiotic therapy, and was discharged from the hospital with improvement of the condition. However, relapse of symptoms occurred after 1 day of discharge from the hospital, prompting readmission. After an additional 7-day course of antibiotic therapy, the patient recovered completely with no recurrence during 1-month follow-up.

**Conclusion:**

Staged diagnosis and individualized therapeutic measures are key to the prognosis of this disease. Despite advances in therapeutic techniques, the risk of relapse is still high, and a comprehensive assessment of infection control is recommended for discharge from the hospital, with intensive follow-up to improve the prognosis.

## Introduction

Emphysematous pyelonephritis (EPN) is an acute fulminant infectious disease characterized by diffuse renal parenchymal necrosis with pneumatosis of the renal parenchyma, renal collecting system, and perirenal interstitial space (1). EPN was first reported in cases by Kelly and Mac Cullum in 1898, and was formally named EPN by Schultz and Klorfein in 1962 (2). The disease is clinically rare and is usually seen in patients with poorly controlled diabetes mellitus, as well as in those with urinary tract obstruction, urinary tract infections, and immunocompromised individuals (3). The disease has an acute onset, rapid progression, and high mortality rates, and requires early and definitive diagnosis, prognosis of severity based on clinical classification, and timely and effective treatment to significantly improve the prognosis. In this report, a rare case of massive emphysematous pyelonephritis combined with short-term relapse is reported through detailed records of disease progression and regression, particularly clear and complete imaging records, with the aim of providing reference for the diagnosis and treatment of this disease.

### Case description

**Figure 1:**
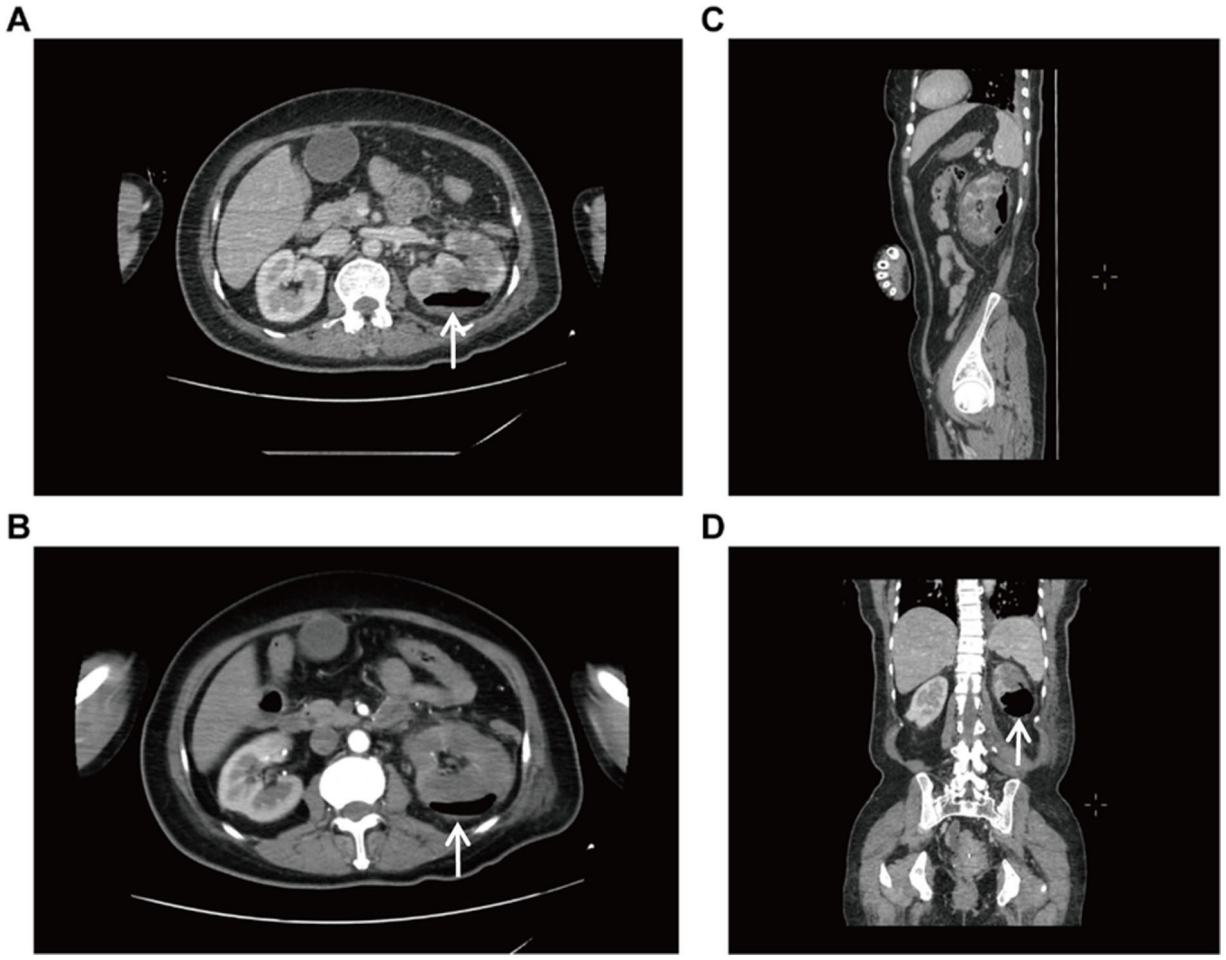
Enhanced CT of the whole abdomen. **(A)** Transverse view (venous phase), increased volume of the left kidney with internal gas shadows. **(B)** Transverse view (arterial phase), showing a patchy hypodense shadow and gas accumulation of the left kidney. **(C)** Sagittal view. **(D)** Coronal view, massive emphysema.

A 56-year-old female patient presented to our hospital on January 28, 2025 with left lower abdominal pain for 5 days. She had a history of diabetes mellitus for more than 10 years, and had been taking metformin and subcutaneous insulin injection at bedtime for a long time to control her blood glucose, with a fasting blood glucose of about 11 mmol/L. The patient occurred left lower abdominal pain with intermittent vomiting without obvious triggers, no chills and high fever, no hematuria and urinary tract irritation 5 days before admission. And she was admitted to an outside hospital for CT scanning of the whole abdomen: the left kidney low density foci with gas density shadow, the left pelvis and calyces of the left kidney and the left ureter intermittent dilatation,suggesting emphysematous pyelonephritis (EPN) and perirenal infection. She received empirical antibiotics (cefoperazone and moxifloxacin) with poor response. Upon admission to our hospital, physical examination demonstrated percussion pain in the left renal region and ureteral point tenderness. Contrast-enhanced abdominal CT showed an enlarged left kidney with patchy hypodense areas and intraparenchymal gas (Figure 1), with the largest gas collection measuring 51 × 42 × 15 mm in orthogonal diameters on venous-phase images, along with perirenal exudation, consistent with EPN. Laboratory examination on admission: WBC 8.03 × 10^9^/L, neutrophil 6.59 × 10^9^/L, PLT34 × 10^9^/L, ultrasensitive CRP 281.68 mg/L, procalcitonin 30.4 ng/mL, fasting blood glucose 12.2 mmol/L, SCr130.1 μmol/L. Urine routine: urine RBC 3+, urine protein 2+, urine glucose 4+, leukocyte esterase 1+. Diagnosis: 1. Acute emphysematous pyelonephritis (left), 2. diabetes mellitus.

**Figure 2:**
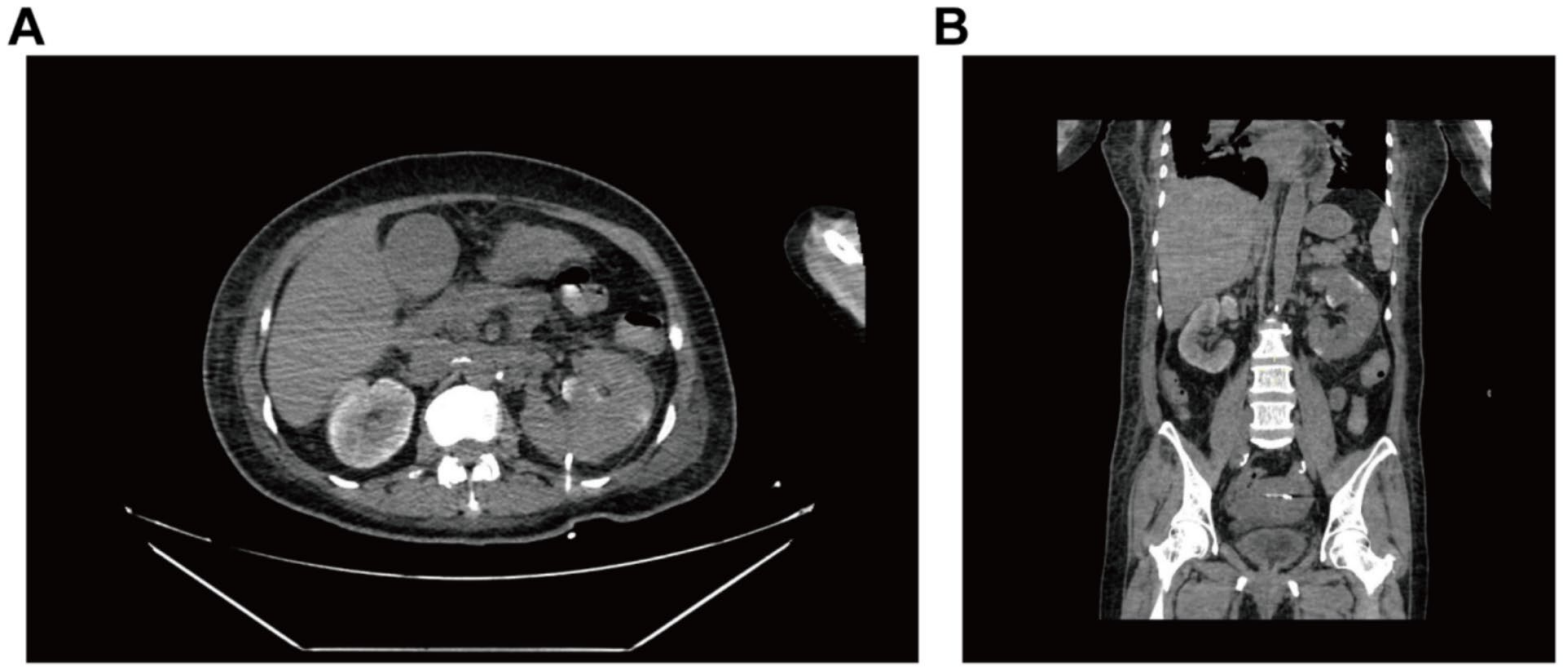
CT-guided whole-abdomen CT plain scan reviewed 2 days after renal puncture and drainage. Both **(A)** axial and **(B)** coronal images demonstrate mild left renal enlargement with perirenal exudation complete resolution of previously seen pneumatosis.

After multidisciplinary consultation, CT-guided percutaneous drainage of the left kidney was performed (Supplementary Figure S1), yielding gas and dark red fluid. Two sets of blood cultures identified gram-negative bacilli, confirming sepsis secondary to EPN, likely due to *Escherichia coli*. The patient was given treatment of anti-infection with meropenem and blood glucose control. On the 2nd postoperative day, the left renal puncture drainage tube was in position and drained dark red fluid, and the examination was repeated: WBC 9.32 × 10^9^/L, neutrophil 8.03 × 10^9^/L, ultrasensitive CRP 269.15 mg/L, calcitonin 61.49 ng/mL, B-type brain natriuretic peptide 8,490, and creatinine 161.7 μmol/L. Besides, hypoxemia (SpO₂ 91% on 6 L/min O₂) raised concern for respiratory failure. Comprehensive treatment with meropenem (1 g, IVD, q8h) for anti-infection, insulin pump for glycemic control, and furosemide for diuresis to improve cardiac function was given. Repeat CT plain scan of the whole abdomen demonstrated reduced gas but persistent perirenal inflammation (Figure 2). On postoperative day 5, the drainage volume decreased and presented as pale sanguineous fluid. Blood cultures (aerobic) identified *Escherichia coli*. The current antimicrobial therapy was continued in accordance with the susceptibility profile (see Supplementary Table S1) with close monitoring of body temperature and serial inflammatory markers.

**Figure 3:**
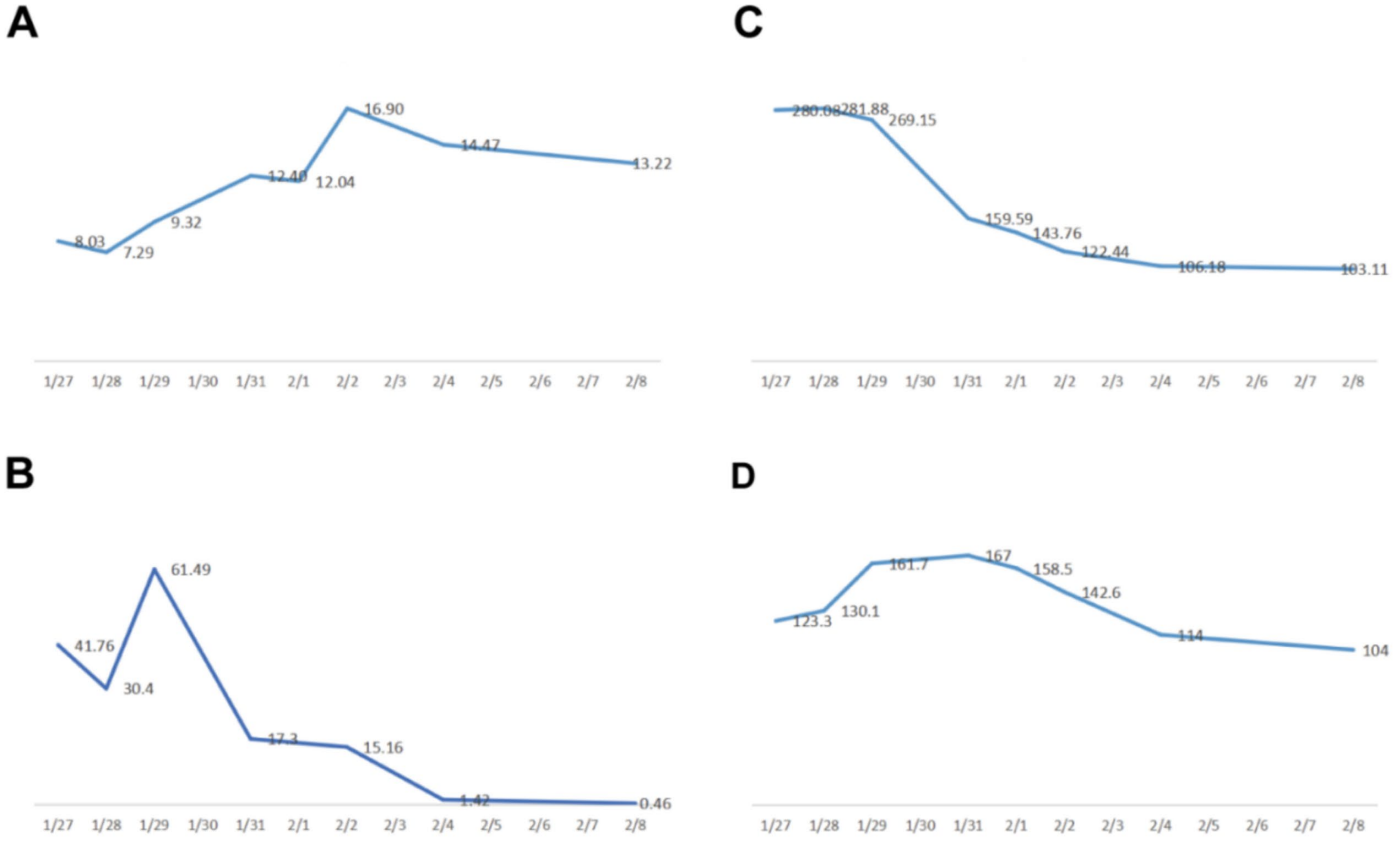
Plot of changes in both blood infection indicators and renal function. **(A)** white blood cells (×10^9^/L). **(B)** procalcitonin PCT (ng/mL). **(C)** ultrasensitive CRP (mg/L). **(D)** creatinine (μmol/L).

**Figure 4:**
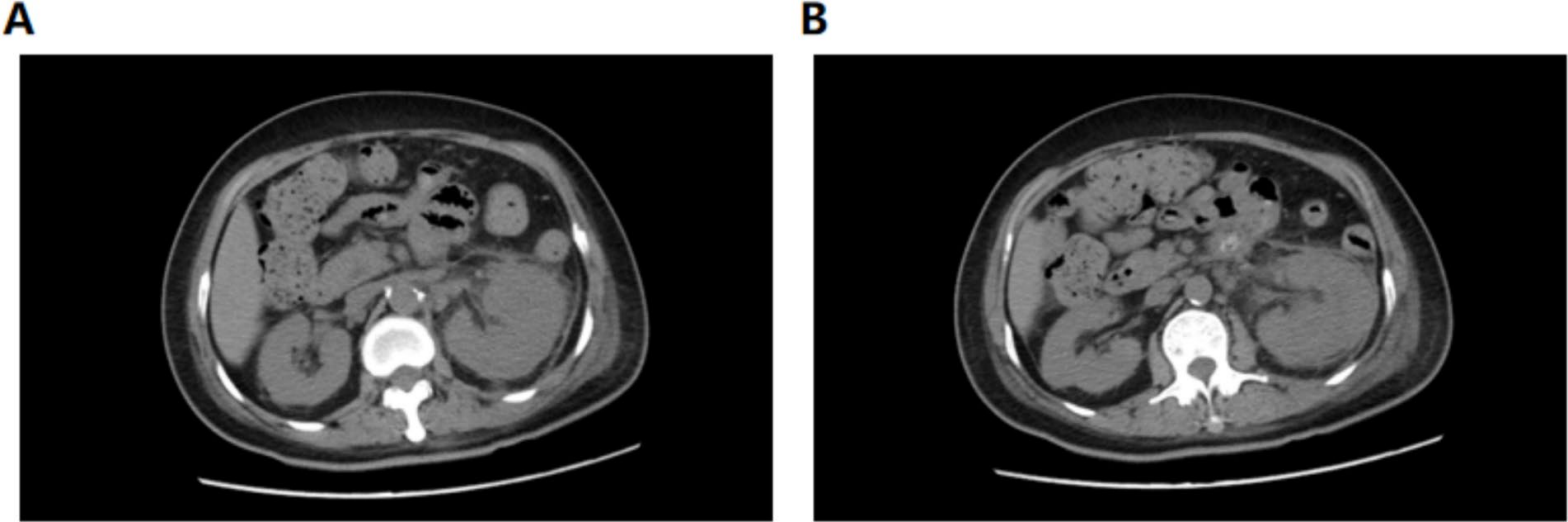
CT plain image of the lower and middle abdomen on 2nd admission review.

On postoperative day 10, renal ultrasound revealed localized renal sinus separation and minimal perirenal exudation in the left kidney. The patient demonstrated significant clinical improvement with marked reduction in fever peaks (Supplementary Figure S2), declining inflammatory markers (Figure 3), improved renal function, along with three consecutive days of drainage output consisting of pale yellow fluid. The drainage catheter was subsequently removed. Antimicrobial therapy was de-escalated from meropenem to cefoperazone-sulbactam (3 g, IVD, q8h). After 3 additional days of treatment with maintained apyrexia (body temperature consistently <37.2 °C) and satisfactory infection control, the patient was discharged with marked clinical improvement. The discharge instructions primarily comprised: (1) oral faropenem (0.2 g, PO, q8h) for antimicrobial therapy; (2) a scheduled follow-up outpatient visit in 2 week; and (3) strict monitoring and control of blood glucose levels. However, she was readmitted 1 day post-discharge with the return of symptoms including flank discomfort, fever (Tmax 38.5 °C) and episodic vomiting. She was investigated in the middle and lower abdominal scans: largely inflammatory changes in the left kidney and ureter (Figure 4), with hsCRP of 101.5 mg/L and leukocytes of 10.54 × 10^9^/L. Cefoperazone-sulbactam was given to counteract the infection, and she was rechecked 1 week later with hs CRP of 45.1 mg/L and leukocytes of 9.29 × 10^9^/L. The patient was successfully discharged on February 19. During the subsequent one-month follow-up period, she maintained no chills and fever, no nausea and vomiting, and no obvious back pain.

## Discussion

Emphysematous pyelonephritis (EPN) is a life-threatening suppurative infection affecting the renal parenchyma and perirenal tissues, predominantly occurring in patients with poorly controlled diabetes mellitus (approximately 95% of cases) (4, 5). The primary causative pathogen is *Escherichia coli*, accounting for nearly 70% of reported cases, while other pathogens include Klebsiella and Aspergillus (1, 4). Our patient was a middle-aged woman with long-standing poorly controlled diabetes. The underlying pathogenesis likely involved a multifactorial process: uncontrolled diabetes leading to diabetic nephropathy and subsequent impairment of renal tissue perfusion, combined with hyperglycemia creating an optimal microenvironment for proliferation of gas-forming pathogens, ultimately culminating in necrotizing gas-producing infection (6). While historical data from the last century reported mortality rates as high as 78% (7–9), contemporary management has significantly improved outcomes. And early diagnostic classification and implementation of personalized multimodal therapeutic strategies now recognized as critical determinants of prognosis.

EPN often presents with nonspecific early clinical manifestations including fever, flank pain, and vomiting. Without prompt diagnosis and effective treatment, patients may rapidly progress to sepsis and even multiple organ dysfunction syndrome. The diagnosis of EPN is mainly reliant on imaging tests, of which CT is the gold standard with an accuracy rate of up to 100%, and abdominal ultrasonography and abdominal plain radiography (KUB) have an accuracy rate of are only 69 and 65%, respectively (1). Ultrasonography shows strong glomerular echoes, which affect observation and characterization due to the presence of a large amount of gas; perirenal pneumatosis on X-ray plain film is difficult to distinguish from intestinal pneumatosis; CT can determine the size and extent of intrarenal and perirenal emphysema, the extent of renal parenchymal destruction and the presence of urinary tract obstruction (10). Based on the CT manifestations, there are currently two methods of EPN staging. The classification of Wan (7): type I is renal necrosis with the presence of gas but no pus; type II is renal parenchyma with the presence of gas and pus in the renal parenchyma, perirenal space, or collecting system. The other is the classification of Huang and Tseng (11), Type I: gas in the collecting system only; Type II: gas in the renal parenchyma but not extending into the perirenal; Type IIIA: gas or abscess extending into the perirenal space; Type IIIB: gas or abscess extending into the pararenal space; Type IV: bilateral EPN or isolated renal EPN.

The classification system of Huang and Tseng (Supplementary Figure S3) has been widely adopted in clinical practice to guide therapeutic decision-making. Multiple retrospective studies (4, 5, 8, 12–14) have demonstrated that: (1) Type I and II patients can be effectively managed with medical therapy alone, supplemented by percutaneous catheter drainage (PCD) when necessary; (2) Type III patients with fewer than two risk factors (thrombocytopenia, acute kidney injury, altered mental status, or shock) may benefit from surgical drainage combined with medical treatment; (3) Type III patients presenting with ≥2 risk factors typically require nephrectomy as the optimal intervention; while (4) Type IV cases demand particularly nuanced management, where bilateral PCD should be attempted initially for renal preservation, with nephrectomy prepared as a contingency measure.

EPN is characterized by rapid progression and high morbidity, necessitating early diagnosis and prompt intervention to improve the prognosis and reduce the mortality rate. In this case,he patient suffered from type 2 diabetes mellitus for more than 10 years, with poor glycemic control, and the characteristic change of gas accumulation in the left kidney tissue was evident on CT. Based on the characteristic CT imaging findings (Supplementary Figure S4), which demonstrated extension of gas and exudate into the perirenal space (the area between the renal capsule and renal fascia, without extension beyond the renal fascia or into adjacent tissues such as the psoas muscle), this case was classified as Huang-Tseng type IIIA emphysematous pyelonephritis. Laboratory tests revealed significant infectious indicators. Initial management comprised aggressive supportive therapy including: (1) empirical broad-spectrum antimicrobial coverage, (2) intensive glycemic control, and (3) electrolyte rebalancing. Following multidisciplinary team evaluation, the patient’s high-risk status was confirmed by the presence of two critical prognostic factors: thrombocytopenia and acute kidney injury. Consequently, a combined therapeutic approach was implemented, consisting of ultrasound-guided percutaneous left renal drainage coupled with culture-directed intravenous antibiotic therapy, in accordance with current management guidelines for type IIIA disease with moderate risk profile. Subsequent blood cultures confirmed *Escherichia coli* as the causative pathogen, prompting continued meropenem therapy. After 2 weeks of treatment, the patient achieved clinical stability with controlled infection and glycemic levels, and was discharged on oral antibiotics. However, the infection relapsed shortly thereafter, necessitating readmission. We propose that the early relapse likely resulted from incomplete eradication of the infection focus. The initial CT scan showed a massive emphysematous lesion with significant tissue necrosis. Although drainage and antibiotics alleviated the acute infection, bacteria may have persisted within the devitalized renal tissue. The complex structure of the necrotic area could have sheltered microorganisms from both antibiotics and immune clearance. When drainage was stopped and antibiotics were de-escalated, these residual bacteria likely proliferated, leading to symptom recurrence. This case illustrates the difficulty in achieving complete sterilization of extensively necrotic tissue, even after apparent clinical improvement. The patient continued one-week antimicrobial therapy and supportive care with a favorable outcomes ultimately. It also highlights that EPN progresses rapidly and is prone to recurrence if infection is not fully controlled before discharge. And it is more necessary to be alert to the fact that the persistent infection may evolve into septicemia or even death. Based on the lessons from this case, we now recommend a more stringent set of criteria before considering discharge for similar conditions. We emphasize the necessity of confirming not just clinical stability but also objective evidence of adequate source control before discharge. Specifically, we recommend ensuring: (1) sustained clinical stability (afebrile for >48 h without antipyretics), (2) consistent downward trend of inflammatory markers (particularly PCT and CRP), (3) imaging follow-up (ultrasound or CT) confirming clinical improvement, (4) evidence of adequate source control (e.g., minimal and clear drainage output if a drain is in place), and (5) stable glycemic control. Importantly, these parameters should be viewed collectively rather than in isolation, with particular attention to the correlation between biochemical and radiological improvement. We believe this comprehensive approach helps prevent premature discharge and reduces relapse risk.

Furthermore, this case must be understood within the broader clinical spectrum of EPN. As illustrated by a recent report from Trang et al. (15), in which EPN progressed to life-threatening pneumomediastinum. While our patient presented with a massive yet locally contained infection that later relapsed, their case demonstrates that inadequate control of EPN can lead to far more than localized recurrence—it may result in gas dissemination along tissue planes with catastrophic consequences. This comparison importantly reinforces the critical need for both complete source control to prevent relapse and continued vigilance to detect atypical, severe complications.

In summary, emphysematous pyelonephritis is a rare clinical and life-threatening infectious disease. Its clinical manifestations lack specificity, and the diagnosis needs to be combined with provisional and imaging features. It is imperative to improve inflammatory indicators, abdominal CT examination and pathogenetic testing at an early stage. Current management combines foundational therapies (broad-spectrum antibiotics, glycemic control, fluid resuscitation) with individualized interventions guided by CT classification and risk stratification to improve the prognosis. Recent advances in diagnostic and therapeutic techniques have dramatically improved EPN outcomes. While mortality rates reached as high as 78% (7, 9) in historical cohorts prior to the late 1970s (pre-CT era), contemporary management strategies have reduced mortality to approximately 13% (16, 17) based on modern meta-analyses encompassing over 1,000 patients. Despite this remarkable improvement, EPN mortality remains substantially higher than that of uncomplicated pyelonephritis. Future studies should focus on optimizing stratified management strategies for high-risk patients, as well as exploring novel antimicrobial agents and supportive treatment options to further reduce the morbidity and mortality of EPN and improve long-term prognosis.

## Data Availability

The original contributions presented in the study are included in the article/Supplementary material, further inquiries can be directed to the corresponding author.
