# Supplementary material for: Case Report: Massive emphysematous pyelonephritis with short-term relapse
Source: Front Med (Lausanne). 2025 Oct 20;12:1660972. doi: 10.3389/fmed.2025.1660972 (PMC12580336; doi:10.3389/fmed.2025.1660972)
Supplement: Supplementary file 1 [file Data_Sheet_1.docx]

## Supplementary Figures


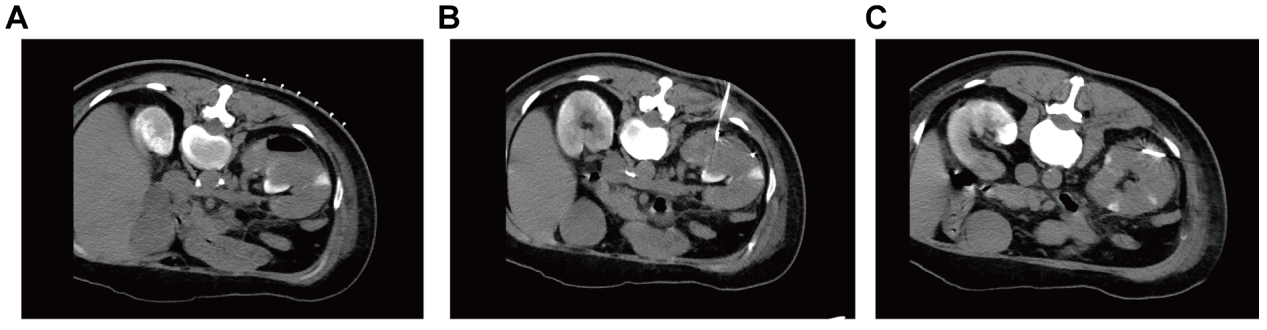


**Figure S1** CT-guided renal puncture drainage.(**A**) before, (**B**) during, and (**C**) after puncture


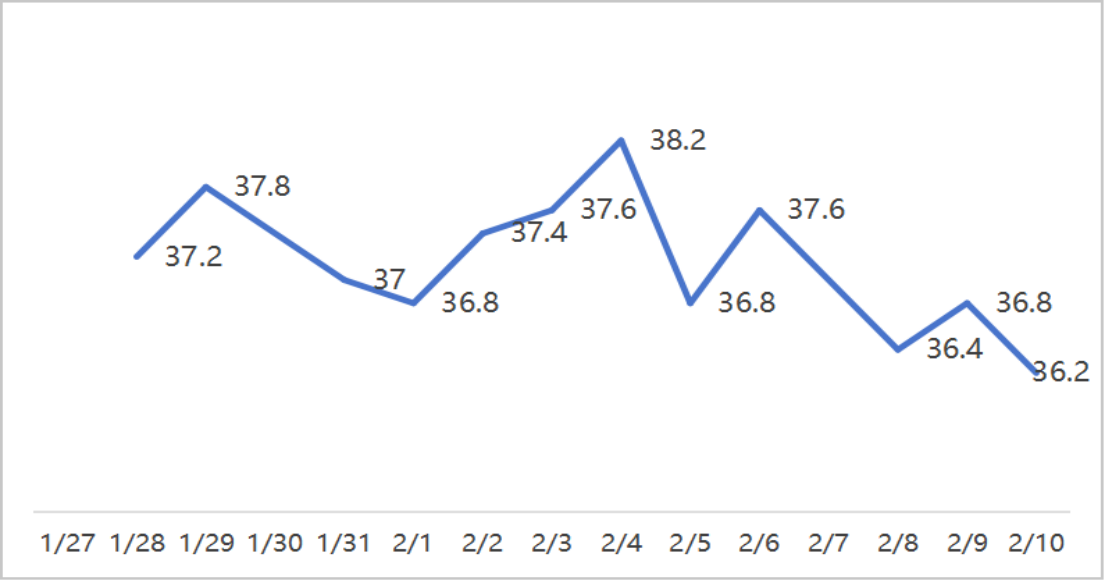


**Figure S2** Plot of the patient's temperature changes over the 2 weeks of admission to the hospital


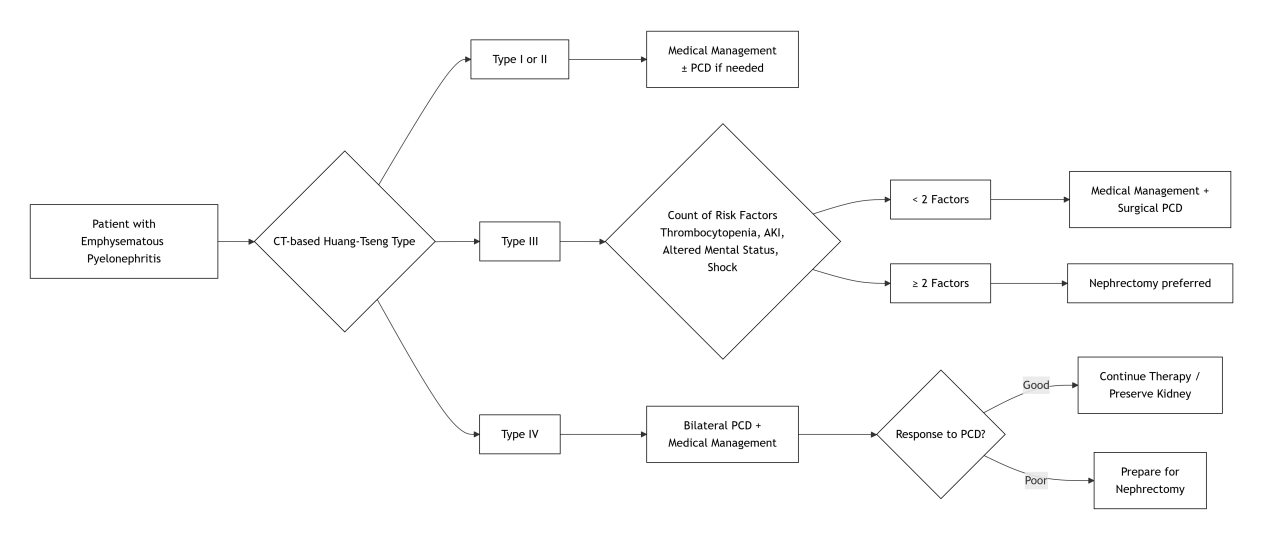


### Figure. S3 **Management Algorithm for Emphysematous Pyelonephritis (Based on Huang–Tseng Classification).**

**
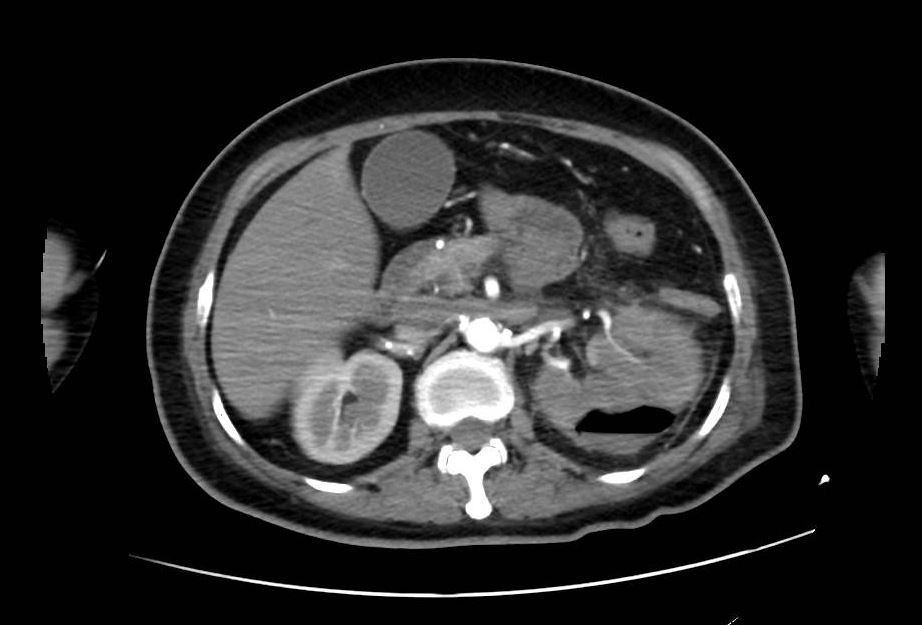
**

### Figure. S4 The extent of gas and exudate diffusion into the perirenal space.

## Supplementary Table

**Table S1: Antibiotic Susceptibility Test Results**

| **Antibiotic Code** | **Antibiotic Name** | **Result** | **Susceptibility** | **Reference (S)** | **Reference (I)** | **Reference (R)** |
| --- | --- | --- | --- | --- | --- | --- |
| AMC | Amoxicillin/Clavulanic Acid | 4.0 | S | <=8/4 | 16/8 | >=32/16 |
| TIC | Ticarcillin | >=128.0 | R | <=16 | 32-64 | >=128 |
| TCC | Ticarcillin/Clavulanic Acid | 16.0 | S | <=16/2 | 32/2-64/2 | >=128/2 |
| PIP | Piperacillin | >=128.0 | R | <=8 | 16 | >=32 |
| TZP | Piperacillin/Tazobactam | <=4.0 | S | <=8/4 | 16/4 | >=32/4 |
| CXM | Cefuroxime | 4.0 | S | <=8 | 16 | >=32 |
| CTT | Cefotetan | <=4.0 | S | <=16 | 32 | >=64 |
| CAZ | Ceftazidime | <=0.12 | S | <=4 | 8 | >=16 |
| CPD | Cefpodoxime | 0.5 | S | <=2 | 4 | >=8 |
| CTX | Cefotaxime | <=1.0 | S | <=1 | 2 | >=4 |
| CZX | Ceftizoxime | <=1.0 | S | <=1 | 2 | >=4 |
| SFP | Cefoperazone/Sulbactam | <=8.0 | S | <=16 | 32 | >=64 |
| FEP | Cefepime | <=0.12 | S | <=2 | 4-8 | >=16 |
| ATM | Aztreonam | <=1.0 | S | <=4 | 8 | >=16 |
| DOR | Doripenem | <=0.12 | S | <=1 | 2 | >=4 |
| IPM | Imipenem | <=0.25 | S | <=1 | 2 | >=4 |
| MEM | Meropenem | <=0.25 | S | <=1 | 2 | >=4 |
| AN | Amikacin | <=2.0 | S | <=4 | 8 | >=16 |
| TM | Tobramycin | <=1.0 | S | <=2 | 4 | >=8 |
| TE | Tetracycline | >=16.0 | R | <=4 | 8 | >=16 |
| DO | Doxycycline | 8.0 | I | <=4 | 8 | >=16 |
| MNO | Minocycline | <=1.0 | S | <=4 | 8 | >=16 |
| CIP | Ciprofloxacin | <=0.25 | S | <=0.25 | 0.5 | >=1 |
| LEV | Levofloxacin | 1.0 | I | <=0.5 | 1 | >=2 |
| MXF | Moxifloxacin | 0.5 | S | <=2 | 4 | >=8 |
| TGC | Tigecycline | <=0.5 | S | <=2 | 4 | >=8 |
| CS | Colistin | <=0.5 | I |  | <=2 | >=4 |
| SXT | Trimethoprim/Sulfamethoxazol | <=20.0 | S | <=2/38 |  | >=4/76 |

**Legend:**

**S:** Susceptible

**I:** Intermediate

**R:** Resistant

**MIC:** Minimum Inhibitory Concentration (µg/ml)
